# 5-alpha Reductase inhibitors and risk of male breast cancer: a systematic review and meta-analysis

**DOI:** 10.1590/S1677-5538.IBJU.2017.0531

**Published:** 2018

**Authors:** Jiamin Wang, Shankun Zhao, Lianmin Luo, Ermao Li, Xiaohang Li, ZhiGang Zhao

**Affiliations:** 1 Department of Urology & Andrology, Minimally Invasive Surgery Center, Guangdong Provincial Key Laboratory of Urology, The First Affiliated Hospital of GuangZhou Medical University, Guangzhou, Guangdong, China

**Keywords:** 5-alpha Reductase Inhibitors, Breast Neoplasms, Male, Meta-Analysis as Topic

## Abstract

**Objective:**

To assess the relationship between 5α-reductase inhibitors (5ARIs) and the risk of male breast cancer (MBC).

**Material and Methods:**

We systematically searched Medline via PubMed, Embase and the Cochrane Library Central Register up to May 2017 to identify published articles related to 5ARIs and the risk of MBC.

**Results:**

Summary effect estimates were calculated by a random-effect model, and tests for multivariable-unadjusted pooled risk ratios (RR) and heterogeneity, as well as the sensitivity analyses were conducted to assess publication bias. All four studies were conducted in a quality assessment according to the Newcastle Ottawa Scale system. The strength of association between 5ARIs and the prevalence of MBC was evaluated by using summarized unadjusted pooled RR with a 95% confidence interval [CI]. Four studies involving 595.776 participants, mean age range from 60 to 73.2 years old, were included in a meta-analysis, which produced a summary unadjusted RR of the risk of MBC for the treatment of 5ARIs of 1.16 (95% CI 0.85-1.58, P=0.36) and the multivariable-adjusted RR is 1.03, (95% CI 0.75-1.41, p=0.86). There was no heterogeneity among included studies (I^2^=0%, P=0.49). Estimates of total effects were generally consistent with the sensitivity.

**Conclusion:**

We did not observe a positive association between the use of 5ARIs and MBC. The small number of breast cancer cases exposed to 5ARIs and the lack of an association in our study suggest that the development of breast cancer should not influence the prescribing of 5ARIs therapy.

## INTRODUCTION

Five-alpha Reductase Inhibitors (5ARIs) finasteride and dutasteride are the competitive and specific inhibitors of 5α-reductase, an enzyme involved in the conversion of testosterone to 5α-dihydrotestosterone (DHT), which is the main androgen involved in the pathogenesis of benign prostatic hyperplasia (BPH) and androgenetic alopecia (AGA; male-pattern hair loss). These drugs act to prevent the transformation process of DHT to slow enlargement of the prostate, and to reduce the potentiation of AGA (1–4). Finasteride selectively inhibits the type 2 5AR enzyme, while Dutasteride is a new generation of 5ARIs that inhibits both type 1 and type 2 5AR enzymes (5). The type 1 isoform is found predominantly in extra-prostatic tissues, such as the skin and liver, whereas type 2 is the isoform found predominantly in normal and hyperplastic prostate tissue (6).

Five-alpha Reductase Inhibitors have been investigated predominantly for their effects on BPH associated lower urinary tract symptoms (LUTS) and for the treatment of AGA on account of abundant 5ARI activities in the prostate and skin. These drugs have been widely used in clinical practice. There have been 50 worldwide case reports of male breast cancer (MBC) in BPH patients aged from 54 to 88 years (mean age 71 years old), who received 5 mg finasteride until 2009, including twenty-seven cases that occurred after finasteride treatment for a minimum of 1 year. There has been an increase in the number of case reports as pointed out in a recent report of Medicines and Health care products Regulatory Agency (MHRA). Patient information issued by the US Food and Drug Administration (FDA) for finasteride includes the following statements: “In rare cases, male breast cancer has been reported” and “The relationship between long-term use of finasteride and male breast neoplasia is currently unknown” (7–9). There has been increasing concern about the possibility of MBC on 5ARIs use in recent years.

MBC is a rare condition, comprising only approximately 1% of all breast malignancies (10). Owing to the rarity of the disease, obtaining a clear picture of risk factors is tremendously challenging, and its etiology remains elusive. Genetic risk factors, including relations with familial history and BRCA gene mutations (11), are well established. Investigations have also reported high risks among patients with Klinefelter syndrome (condition characterized by 46-XXY karyotype and relative excesses of estrogens in relation to androgens) (12, 13). Several studies indicated that MBC was highly related to obesity (14–20), physical inactivity (14, 19, 20), exogenous hormone use (21–24), and diabetes (17–25). Collectively, these findings emphasize the need for assessing the roles of endogenous hormones in relation to MBC. High levels of both estrogens and androgens have been implicated in female breast cancer (26). Therefore, we conducted this study to summarize the available evidences with the purpose of assessing the relationship between 5ARIs and the prevalence of MBC. To the best of our knowledge, this is the first meta-analysis about this topic.

## MATERIALS AND METHODS

### Literature Search Strategy and Selection Criteria

Literature regarding meta-analysis studies was searched without language restriction up to May 2017, in databases including Embase, Cochrane library and Medline via PubMed. Additionally, we restricted the search to human participants. We used subject headings and keywords for each electronic databases. The following search terms were used: 5-alpha-reductase inhibitors, finasteride, dutasteride, 5ARI, Breast Neoplasms, Breast Tumors, Breast Cancer, Mammary Cancer and Malignant Tumor of Breast. We searched for additional relevant studies by examining the reference lists of the articles and published reviews.

On the basis of patient, intervention, comparison, outcome and study design (PICOS) (27), studies were included if it assessed men with AGA and BPH (P); the use of ARIs (I); comparing the drug with an attention placebo control group (C); and patient outcomes of MBC (O). Eligible trials were RCTs or prospective trials that compared 5ARIs with placebo for AGA or BPH. In addition, the included studies provided the incidence of MBC or the number of men with it or sufficient data (e. g, estimates, standard error, and P value) to calculate them. Studies of combination therapy of ARIs plus alpha-blockers for BPH were not included. Review articles, meeting abstracts, editorials, case reports, and commentaries were also excluded if they have been useful for background information. For each potential included study, two investigators independently carried out the selection evaluation, data abstraction, and quality assessment. Disagreements were resolved by discussion or in consultation with a third author when two investigates independently selected studies for inclusion in this study.

All these information were recorded in a standardized form and the following data were sought from each study: year of publication, first author's name, ethnicity, diagnostic method, type of investigation, exact number of participants both in case and control groups, and relative risks as well. We assessed the quality of selected cohort studies and case-control studies according to the Newcastle-Ottawa quality assessment scale (NOS) (28); the instrument consists of three domains indicating the study quality as: selection (4 points), comparability (2 points) and outcome (3 points) for a total score of 9 points (with 9 representing the highest quality). Studies scoring 7-9 points, 3-6 points and 0-3 points were identified as high, moderate and low quality, respectively.

Data were analyzed using the Review Manager 5.1.2 statistical package (Cochrane Collaboration Software) (29), and the clinical outcomes were reported as risk ratio (RR). The corresponding 95% confidence interval (95% CI) was calculated, considering P values less than 5% (P <0.05). A statistic for measuring heterogeneity was calculated through I^2^ method (25-50% was considered low-level heterogeneity, 50-75% moderate-level heterogeneity and >75% high-level heterogeneity). We carried out an additional analysis using the random-effects model described by DerSimonian et al. (30), to observe if there was statistical heterogeneity found in the meta-analysis. We executed the funnel plot test described by Egger et al. (31) to determine the possibility of any publication bias. For all analyses, a forest plot was generated to display results. After that, subgroup analysis was further carried out by different races, to appraise sources of heterogeneity. In addition, sensitivity analysis was performed with the method of calculating the unadjusted pooled RR by repeating the overall analysis after omitting each study in turn.

## RESULTS

**Figure 1:**
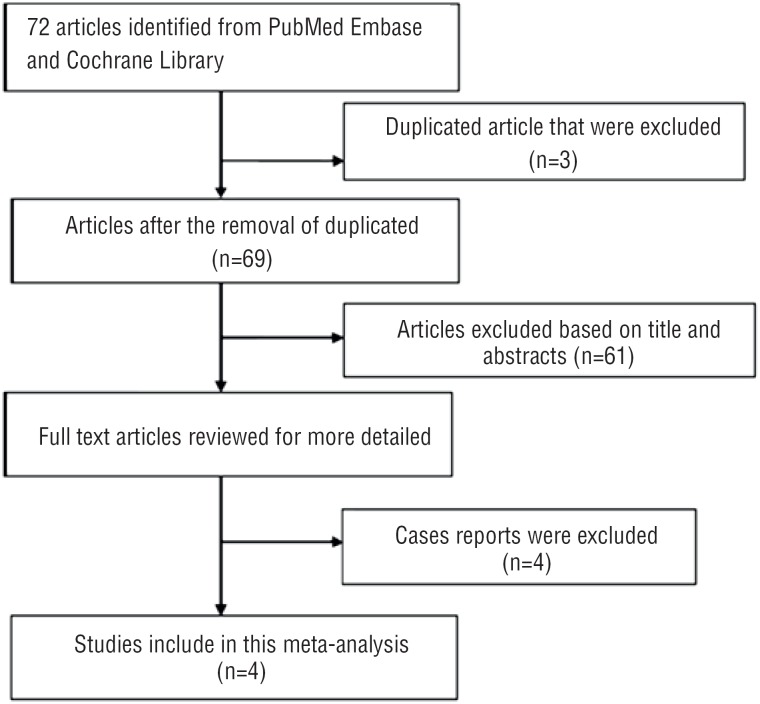
Flow chart of study selection.

The diagram represents the flow of identification and inclusion of trials, as recommended by the Preferred Reporting Items for Systematic reviews and Meta-Analyses (PRISMA) statement (32) (Figure-1). In the first search, 72 references were identified and screened. 61 studies were excluded as unmatched titles and abstracts. By reviewed full text, 4 cases reports and 3 repeated studies were further removed, the lasted 4 studies were what we needed (33–36).

The baseline characteristics of the included studies are shown in Table-1. Included studies (2 case-control and 2 cohort studies) were published between the years of 2013 to 2017, accounting for more 558.281 individuals and 37.495 MBC cases were examined. Information on exposure and outcome was completely gained from cancer registry and official clinical practice research. All these studies were carried out in the following races: European (n=3), and American (n=1).

**Table 1:** Characteristics of the included studies.

Study	Year	Country	Race	Study design	Study group case/total	Control group case/total	Mean age (years)		International Classification of Diseases	Unadjusted RR, 95% CI	Adjusted RR, 95% CI
							Study group	Control group			
Robinson et al. (33)	2015	Sweden	European	cohort study	4/36620	75/545293	72	70	ICD-50	0.79(0.29-2.17)	0.65 (0.32-1.31)
Hagberg et al. (34)	2017	UK	European	case-control	10/48	58/478	73.2±9.9	73.1±9.9	protocol numbers 15_086 and 15_124	1.91 (0.90-4.03)	1.52 (0.613.80)
Duijnhoven et al. (35)	2014	UK	European	case-control	17/398	150/3930	71±11.0	70.9 ±10.8	NA	1.12 (0.67-1.88)	1.08 (0.62-1.87)
Bird et al. (36)	2013	USA	USA	cohort study	15/429	278/8580	60(51-68)	60(51-68)	ICD-9-CM 175.x	1.08 (0.64-1.84)	1.12 (0.65-1.93)

**NA** = not available

Two of the four studies which were evaluated by the Newcastle-Ottawa Scale (NOS) all are of the high level quality, ranging from 7 to 9. The results of quality assessment are summarized in Table-2.

**Table 2:** New castle-Ottawa Scale (NOS) assessment of the quality of the cohort and case-control studies.

Study	Selection				Comparability		Exposure/Outcome			Total scores
	1	2	3	4	5^A^	5^B^	6	7	8	
Robinson et al. (33)	–	✰	–	✰	✰	–	✰	✰	✰	6
Hagberg et al. (34)	✰	✰	–	✰	✰	✰	✰	–	✰	7
Duijnhoven et al. (35)	✰	–	✰	✰	✰	✰	–	✰	✰	7
Bird et al. (36)	–	✰	✰	–	✰	✰	–	✰	✰	6

For cohort studies: 1 representativeness of the exposed cohort; 2 selection of the non-exposed cohort; 3 ascertainment of exposure; 4 demonstration that outcome of interest was not present at start of study; 5 comparability of cohorts on the basis of the design or analysis; 6 assessment of outcome; 7 was follow up long enough for outcomes to occur; 8 adequacy of follow-up of cohorts.

For cases-control study: 1 case definition adequate; 2 representativeness of the cases; 3 selection of controls; 4 definition of control; 5 comparability based on design or analysis; 6 ascertainment of exposure; 7 same method of ascertainment for cases and controls; 8 non-response rate.

A Studies that controlled for age received one score

B Studies that controlled for other important confounders received an additional score

**Figure 2:**
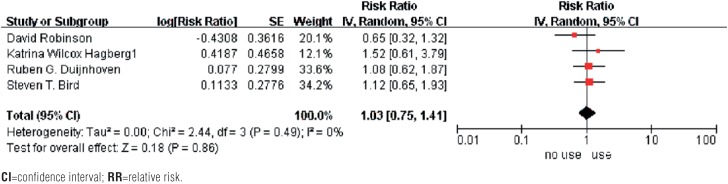
The multivariable adjusted RR of meta-analysis

**Figure 3:**
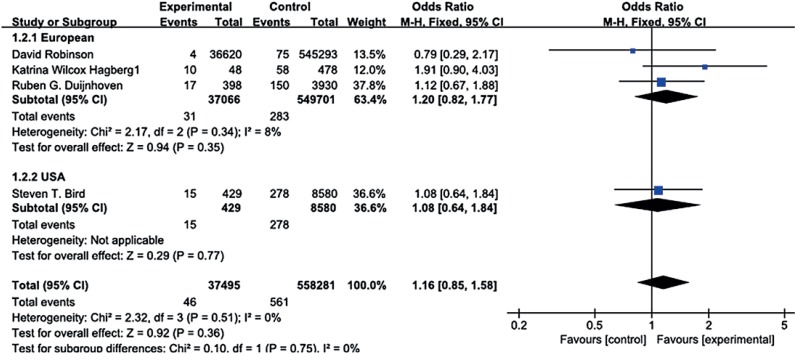
The result of multivariable unadjusted RR and subgroup analysis of meta-analysis.

The analysis showed a non-significant increase risk of MBC for exposure to 5ARIs compared to a nonexposure (RR=1.16, 95% CI 0.85-1.58, P=0.36), and the multivariable-adjusted RR is 1.03, (95% CI 0.75-1.41, P=0.86). There was no evidence of heterogeneity (I^2^=0%, P=0.49), which made us calculated with random effects model (Figures 2 and 3).

Sensitivity analysis was utilized to detect the influence of each study on the pooled RR by repeating the meta-analysis, while omitting 1 single study each time. The sensitivity analysis for the risk of MBC on use of 5ARIs ranged from 1.04 (95% CI: 0.75-1.44, P=0.83) to 1.21 (95% CI: 0.861.71, P=0.27), shown in Table-3, demonstrating that no individual study significantly affected the pooled RR. Thus, sensitivity analysis showed that our results were reliable and no single study dominated the combined RR. We performed stratified analyses by race (Figure-3), which showed the studies from European (RR 1.20, 95% CI 0.82-1.77, P=0.35) compared with studies from American (RR 1.08, 95% CI 0.64-1.84, P=0.77).

**Table 3:** Sensitivity analysis after each study was excluded by turns.

Study omitted	RR (95% CI) for remainders	Heterogeneity	
		I^2^ (%)	P
Robinson et al. (33)	1.16 [0.81,1.65]	0	0.81
Hagberg et al. (34)	0.98 [0.69, 1.37]	0	0.44
Duijnhoven et al. (35)	1.00 [0.65, 1.55]	16	0.30
Bird et al. (36)	0.99 [0.64, 1.51]	13	0.32

**CI**=confidence interval; **RR**=relative risk.

**Figure 4:**
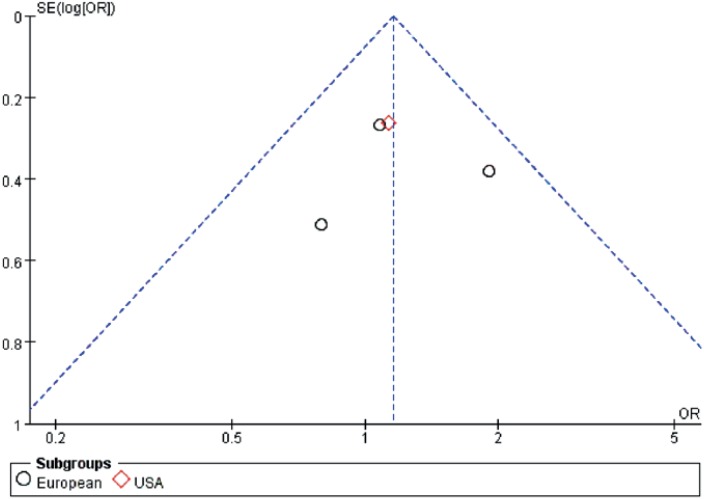
Funnel plot.

In our assessment of publication bias, funnel plots showed balance, with points distributing around the verticals, indicating no obvious publication bias (Figure-4).

## DISCUSSION

To the best of our knowledge, this is the first meta-analysis which considers the effect of treatment of 5ARIs on the risk of MBC. From the selected articles, we clearly founded that MBC appears to be unrelated to the treatment of 5ARIs. This result was consistent with the study of Hagberg et al. (34) that collected the entire male population of Sweden who have used 5ARIs in all registers; the conclusion was similar to ours, but there was an increased risk of MBC on men who had undergone a transurethral resection of the prostate (TUR-P). The small number of cases with male breast cancer and the lack of information on some known risk factors for male breast cancer such as family history, obesity, and exposure to radiation are the biggest limitation of this study. Neither short-term nor long-term treatment was associated with a significantly increased risk of MBC (33). Though the author did not use data from cancer registries for case ascertainment where are often perceived as the gold standard, CPRD however also has a proven high reliability in general as well as for cancer diagnoses. Bird et al. (36) and Duijnhoven et al. (35) declared a similar conclusion with us, they did not observe a positive association between 5ARIs and MBC, even in 1 to 3-year time frames. The lower incidence of MBC makes it a limitation, that is, it is a difficult cancer to study. They need to obtain cases from an underlying population of more than 68 million.

As the disease incidence is less than 1% of female breast cancer, male breast cancer has not been studied well, and limited information is available regarding the epidemiology, pathogenesis, and treatment (37). We still need to pay sufficient attention to the risk of MBC on use of 5ARIs constantly, though our results suggest that there is no increased risk of breast cancer among men using 5ARIs compared to unexposed men. McConnell et al. (13) reported that four cases of breast cancer were diagnosed in patients who received finasteride alone or in combination with doxazosin. The rate of breast cancer in this trial for men taking Finasteride either alone or with doxazosin was therefore 4 in 1554. It's nearly 200 times of the morbidity of breast cancer. In addition, the results of both case reports and clinical trial results have suggested that treatment with 5ARIs may be associated with MBC (38, 39). An evidence review by the United Kingdom's (UK) national drug agency (12) made a finasteride drug warning label for breast cancer in the UK.

MBC is extremely rare, with an incidence in the general US population of <1%. It tends to be diagnosed at later stages than breast cancer in females, likely because of low awareness on the part of the patient and low suspicion by the physician. Clinical manifestations of MBC include breast mass (seen in 75% of patients), nipple retraction (9%), nipple discharge (6%), skin/nipple ulceration (6%) and Paget's disease of the nipple (1%) (40). Risk factors for male breast carcinoma include BRCA1 andBRCA2 mutations, Klinefelter's syndrome, altered testosterone and estrogen balance, testicular disorders, obesity, carcinoma of prostate and its treatment. Contradictory evidence has been reported in literature about gynecomastia as a risk factor for breast cancer in males with evidence both for and against it (41, 42).

Multifactorial effects are contributory to male breast tumorigenesis. We have no evidence to show that 5ARIs increased the risk of MBC according to our meta-analysis. We suggested that is because BRCA1 andBRCA2 mutations play a major role in the development of MBC.

The BRCA1 and BRCA2 protein products are the key-point in DNA repair and cell cycle check point control (43, 44). They are classified as tumor-suppressor genes as well as maintain genomic stability and control of cell proliferation (43). Mutations in the BRCA1 OR BRCA2 genes result in the cells inability to repair DNA damage, allowing for the accumulation of genetic instabilities that can alter cell-cycle checkpoint control. Dysfunctional checkpoint control enables cells to proliferate and become tumoral. Accordingly, BRCA mutation carriers show higher risk for breast (in both females and males) cancers (45).

The strongest risk factor for MBC is the presence of an inherited BRCA2 mutation. The lifetime risk for breast cancer in a male BRCA2 mutation carrier is 7%, 80-100 times higher than for the general population (45–47). It is estimated that 4-40% of MBC patients carry a mutation in BRCA2 (48–50). However, a precise estimate is limited because few studies have included populations of males who were not already diagnosed with breast cancer. The association between BRCA1 mutations and MBC is not as strong as that seen for BRCA2 mutations. The lifetime risk for breast cancer in a male BRCA1 mutation carrier is just over 1%, and it is estimated that a BRCA1 mutation is present in up to 4% of MBC cases (43, 51, 52). Our study suggested that the development of breast cancer should not influence the prescribing of 5ARIs therapy.

The low incidence of MBC provides limited information on epidemiology, pathogenesis, and treatment to study. What is more, although we did not find any evidence of publication bias by testing funnel plots for obvious asymmetry, there still might be some unpublished studies that would nullify our results.

## CONCLUSIONS

The inadequate information and the relatively short times to onset in these cases make the causal association between MBC and finasteride unlikely. In the future, more epidemiological and clinical studies are required to further explore the association between 5ARIs and risk of MBC.
